# Ten Simple Rules for Reducing Overoptimistic Reporting in Methodological Computational Research

**DOI:** 10.1371/journal.pcbi.1004191

**Published:** 2015-04-23

**Authors:** Anne-Laure Boulesteix

**Affiliations:** Institute for Medical Informatics, Biometry and Epidemiology, Ludwig Maximilians University, Munich, Germany; Whitehead Institute, UNITED STATES

## Introduction

In most scientific fields, and in biomedical research in particular, there have long been many discussions on how to improve research practices and methods. The trend has increased in recent years, as illustrated by the series on “reducing waste,” published in The Lancet in January 2014 [1], or by the recent essay by John Ioannidis on how to make published results more true [2], which echoes his earlier provocative paper entitled “Why most published research findings are false” [3]. One of the important aspects underlying these discussions is that biomedical literature is most often overoptimistic with respect to, for example, the superiority of a new therapy or the strength of association between a risk factor and an outcome. Published results appear more significant, more spectacular, or sometimes more intuitive—in a word, more “satisfactory”—to authors and readers than they actually would if they reflected the truth. Causes of this problem are diverse, numerous, and interrelated. The effects of “fishing for significance” strategies or selective/incomplete reporting are exacerbated by design issues (e.g., small sample sizes, many investigated features) [3] or publication bias [4], to cite only a few of the factors at work.

Research and guidelines on how to reduce overoptimistic reporting in the context of computational research, including computational biology as an important special case, however, are surprisingly scarce. Many methodological articles published in computational literature report the (vastly) superior performance of new methods [5], too often in general terms and—directly or indirectly—implying that the presented positive results are generalizable to other settings. Such overoptimistic reporting confuses readers, makes literature less credible and more difficult to interpret, and might even ultimately lead to a waste of resources in some cases. Here I take advantage of the popular “ten-simple-rules” format [6] to address the problem of overoptimistic reporting in methodological computational biology research, that is papers—termed “methodological papers” here—devoted primarily to the development and testing of new computational methods (intended to be used by other researchers on other data in the future) rather than to the biological question itself or the specific dataset at hand.

## Rule 1: Assess the New Method

If your goal is to present a new method and convince readers to use it, assess this new method. Applying it to data to answer a biological question and obtaining plausible, interesting results is nice. But this is not sufficient to establish that the new method has advantages over existing methods, nor is it adequate in providing trustworthy biological results—since the validity of the computational method has not yet been assessed. It is not impossible—but it is difficult—to both assess a new computational method and address a relevant biological question in the same article. The assessment of the new method may be performed in different ways depending on the context, for example, by conducting simulations, applying the method to several real datasets, checking the underlying assumptions in practical examples, etc. Notably, if there exist competing methods for performing the same task, they should be compared to the new method; see Rule 2 for more details.

## Rule 2: Compare the New Method to the Best

A new method will be useful in practice only if it performs better (see Rule 6 for a discussion of “better performance”) when compared to the best existing methods performing the same task. The new method should not be compared to old methods no longer in use, to obsolete versions of currently used methods, or to good methods with suboptimal parameter settings: comparing the new method to suboptimal competitors will inevitably make it look better than it actually is. This rule is especially important for research topics common in the literature. For example, for supervised classification based on high-dimensional omics data, tens or even hundreds of methods have already been proposed: a new method should not be solely compared to basic methods such as naive Bayes. See [7] for a more in-depth discussion of this problem. Note, however, that recent methods are not always publicly available as user-friendly computer programs, which may make comparison challenging in practice.

## Rule 3: Consider Enough Datasets

To establish that a new method works well in practice, it is important to evaluate its performance using several datasets, just as it is important to evaluate the efficiency of a new drug based on several patients before recommending it for use on other patients [5,8]. With this analogy in mind, it becomes clear that many datasets are needed if one wants to firmly establish the superiority of a new method, and that the question of “how many” is essentially a statistical question [9].

For example, if one compares the performances of two methods—as measured by a normally distributed criterion—on ten datasets, a paired t-test may be used for statistical inference. Beyond the t-test itself, one may also, for example, derive a confidence interval for the difference between the performances of the two methods, apply a multiple testing procedure if more than two methods are compared, or compute the power of the paired t-test to detect a given difference considered relevant by the researcher [9].

In simulations, it will generally not be a problem to consider a (very) large number of datasets, except in cases where the analysis of each dataset is extremely computationally expensive. If one could generate and analyse infinitely many datasets for a given simulation setting, there would be no need to perform a test to assess the difference between the performances of the considered methods: the distribution of this difference would be known. In practice, one should generate and analyse as many datasets as computationally feasible.

For comparisons based on real datasets, however, it may be difficult to find—and have access to—enough adequate example datasets. For topics such as supervised classification based on high-dimensional omics data, numerous well-documented datasets can be found in publicly available databases like ArrayExpress, GEO, and TCGA—to cite only a few. For more complex or recent research questions or data types, however, it may be difficult to apply the new method to more than one or two illustrative datasets.

If the data examples are merely meant as illustrations, which is also fine, it should be stated clearly that they are not intended to be representative of what would happen with similar datasets [8]: in this situation, interpretations of and conclusions on the performance of the new method should be formulated cautiously.

## Rule 4: Do Not “Fish” for Datasets

Example datasets should not be selected just because they yield favorable results for the new method. Similarly, one should not exclude a dataset from the analysis just because it yields bad results. The dramatic consequences in terms of overoptimism of such a “fishing for datasets” strategy have been assessed elsewhere through theoretical modeling and simulations [10] and empirical studies [7]. Ideally, one should define “inclusion criteria” for datasets (e.g., datasets with a particular size or format, on pre-specified diseases, etc.), apply the methods, and report all results.

These inclusion criteria should reflect the intended field of application: if most real datasets have certain features, then the datasets to be selected should also have this feature. For example, it would be unsuitable to include only large datasets in the study if most datasets in the target research field are smaller; such a study may even produce misleading results, since the relative performances of the considered methods may, to some extent, depend on the dataset’s size.

## Rule 5: Think of the No-Free-Lunch Theorem and Report Limitations

No reasonable researcher requires your method to always work better than existing methods. Think of the widely acknowledged “no-free-lunch theorem” [11]. Methods are not characterized by a single criterion; see also Rule 6. Datasets are extremely diverse, and so are the performances of methods when applied to them. Referees are supposed to be reasonable researchers, so they will most likely not prevent the publication of your paper simply because your new method is not perfect in all situations and in all respects. Do not forget that, and interpret your results accordingly.

In particular, report limitations of your method and study. In medical literature—those reports on new medical discoveries obtained with an existing data analysis method described elsewhere—the section on “limitations of the study” is considered crucial. Limitations of the method’s applicability, practical problems, implementation issues, and pitfalls related to the study design should also be stated clearly in a methodological paper. This rule is related to Philip Bourne’s Rule 2 on objectivity in the first ten-rules article [12].

## Rule 6: Consider Several Criteria

Do not become obsessed by a single objective performance criterion, such as, in the case of supervised learning, predictor error. Many other aspects of a new method are important, for example, its computational efficiency, its generalizability, its conceptual simplicity, its lack of sensitivity to the choice of parameters or starting values, and its robustness against the violation of assumptions, to cite only a few. Note that, in computational biology, the ground truth is often unknown in real data applications, which makes the measurement of performance difficult. In these situations, the ability of the new method to uncover the truth can be evaluated using simulations (see Rule 8), and alternative criteria, such as those listed above, can be used to assess the method’s behaviour in real data settings. Considering several criteria naturally reduces overoptimistic reporting because—most often—no method is better with respect to all criteria. Further, such considerations also provide a more complete picture of the method’s performance and utility.

## Rule 7: Validate Using Independent Data

The new method should be evaluated using data that were not used during the development phase. For example, consider the case of a new machine learning method for supervised classification, such as a new variant of support vector machines (SVM). Its prediction error on a real dataset of moderate size is typically measured through cross-validation (CV) techniques. One obtains as many cross-validation estimates of predictor error as considered datasets. Now imagine modifying some of the new method’s characteristics in a trial-and-error process, gradually improving these CV estimates [13]. Ultimately, the CV error estimates could be relatively small as a result of this optimization process and the new method would seemingly work well if evaluated using these datasets: the new method would overfit the datasets used for its development. But this says nothing about the ability of the new method to work well on other, independent datasets—for which the prediction error may be much higher. See also [7] for an empirical study on the potential impact of such optimization mechanisms in practice. For proper evaluation, one has to use other—independent—datasets, which are not examined by the researchers until the new method is fully specified.

Note that this problem is similar to the well-known problem that in machine learning one should not evaluate a prediction rule on the training data on which it was fit. However, here we are concerned with the validation of the methods’ general performance rather than with the validation of results obtained with these methods on a specific dataset. In our example, we consider the evaluation of the general performance of the SVM variant rather than the evaluation of the prediction rule resulting when this SVM variant is applied to a specific training dataset.

## Rule 8: Design Simulations Appropriately

A simulation should ideally encompass different settings (e.g., different data sizes, different correlation structures, etc.), which roughly reflect the type of data encountered in the intended area of application. Simulations should not be limited to artificial datasets corresponding exactly to the assumptions underlying the new method, as this would obviously favor the new method. Other data types should be considered as well. Ultimately, the practical relevance of a simulation depends on the similarity between the considered simulation settings and the real datasets in the area of application. Finally, while interpreting simulation results, one should not forget that simulated datasets represent but a tiny dot in the infinite space of possible parameters and settings, which can be seen as an intrinsic limitation of simulations—needing to be discussed, as stipulated by Rule 5. In practice, this problem can be stressed, for example, through phrases such as “in our simulation setting, we found that…”

## Rule 9: Provide All Information

The new method’s definition, its underlying assumptions, its parameters, the study design, data preparation steps and, last, but not least, implementation issues and computer codes for reproducibility purposes [14] should be carefully reported. Whenever possible, data should be made publicly available so that interested readers can rerun analyses, check results, try alternative analysis strategies, or better compare the study’s results to that of their own study’s. Reporting has been a widely discussed topic in the last few years in biomedical research [15]. We claim that it also deserves attention in the context of methodological computational research. High-quality reporting, including, but not limited to, computational reproducibility through publication of codes and (whenever possible) data, reduces overoptimism and its impact by increasing transparency and allowing readers to better interpret results to counter the potentially overoptimistic statements of the authors.

## Rule 10: Read the Other Ten Simple Rules Articles

Some rules presented in other ten-simple-rules articles are also directly or indirectly related to overoptimistic reporting, for instance, those on writing papers [16], better figures [17], getting published [12], efficient computational research [18], and reproducible research [14].

## Conclusion

Some amount of overoptimism is certainly unavoidable in literature. From a purely statistical point of view, type I error is non-zero even if a test is performed correctly. Correspondingly, one cannot expect literature to be free of false positive research findings.

Of crucial note is that the problem of overoptimism is related to publication policies and publication bias. As long as journal editors and referees reject sound studies on sensible ideas simply because the new method was not vastly superior to existing methods, authors will always have to be somewhat overoptimistic (possibly also including ourselves!). Reducing the so-called publication bias in the context of methodological research is a challenge that still has to be addressed both from an epistemological point of view (what is actually publication bias?) and from a practical/editorial perspective (which reduction measures could be reasonably undertaken by journals?).

To conclude, overoptimistic reporting is a problem with multiple facets. Advice to authors and solutions to reduce overoptimism should go beyond a ten-rules article. However, following the ten simple rules above can have a considerable influence on alleviating the problem of overoptimism in reporting.
